# Late Onset Atypical Pantothenate-Kinase-Associated Neurodegeneration

**DOI:** 10.1155/2013/860201

**Published:** 2013-03-24

**Authors:** Natalie Diaz

**Affiliations:** Los Angeles Biomedical Institute, Harbor-UCLA Medical Center, Box No. 492, 1000 W. Carson Street, Los Angeles, Torrance, CA 90509, USA

## Abstract

*Introduction*. Pantothenate-kinase-associated neurodegeneration (PKAN) is a rare genetic disease and a form of neurodegeneration with brain iron accumulation (NBIA). It most commonly begins in the first two decades of life but should be considered in the differential diagnosis of patients at any age with an atypical progressive extrapyramidal disorder and cognitive impairment. Few late-adult cases have been reported. *Case Report*. A 50-year-old woman presented with a history of progressive dysarthria and dysphagia secondary to orolingual dystonia. Initial work-up was normal. There was no family history. Her initial symptoms were followed by the onset of blepharospasm, cervical dystonia, Parkinsonism, and cognitive impairment. Follow-up MRI four years after presentation revealed the diagnostic “eye-of-the-tiger” sign. Genetic testing confirmed a homozygous missense mutation consistent with the diagnosis of PKAN. *Conclusion*. Although PKAN is a rare genetic disorder most commonly seen in childhood, it should be considered in adult patients with a history of progressive focal dystonia or atypical Parkinsonism. As the radiographic findings are quite characteristic, genetic testing should be performed if the MRI shows evidence of iron accumulation. Optimal treatment strategies are not known, and at the current time therapies should be directed at the specific manifestations of the disease.

## 1. Introduction

Pantothenate-kinase-associated neurodegeneration (PKAN) is a rare, autosomal recessive disorder that most commonly begins in the first two decades of life with progressive extrapyramidal manifestations. It is part of a group of disorders under the umbrella term neurodegeneration with brain iron accumulation (NBIA). Originally called Hallervorden-Spatz Syndrome (HSS) after the two German pathologists who first published descriptions of a progressive extrapyramidal disorder associated with pathological iron deposition in the globus pallidus and substantia nigra pars reticulata [1], it was later renamed NBIA in 2003 to dissociate the disorder from atrocities committed during the second World War. With the discovery of the first genetic mutation found in the pantothenate kinase 2 (PANK2 gene) in 2001 [2], NBIA patients with this mutation were subclassified under the new term pantothenate kinase-associated neurodegeneration (PKAN). NBIA now includes (a) PKAN (also called NBIA1) caused by mutations in the PANK2 gene, (b) PLA2G6-associated neurodegeneration (PLAN, NBIA2) due to phospholipase A2 mutations, (c) neuroferritinopathy due to mutations in the ferritin light chain (FTL) gene, (d) aceruloplasminemia due to a mutation in the ceruloplasmin gene, and (e) sporadic cases of NBIA in which the genetic background has not been identified. PKAN is thought to be the most common form of NBIA and accounts for more than 50% of cases of NBIA [3]. PKAN and PLAN typically have onset in childhood while aceruloplasminemia and neuroferritinopathy typically present at an older age in the fifth and sixth decade. Here we describe a patient who presented in her late fifth decade with symptoms of a childhood onset NBIA disorder.

## 2. Case Report

**Figure 1:**
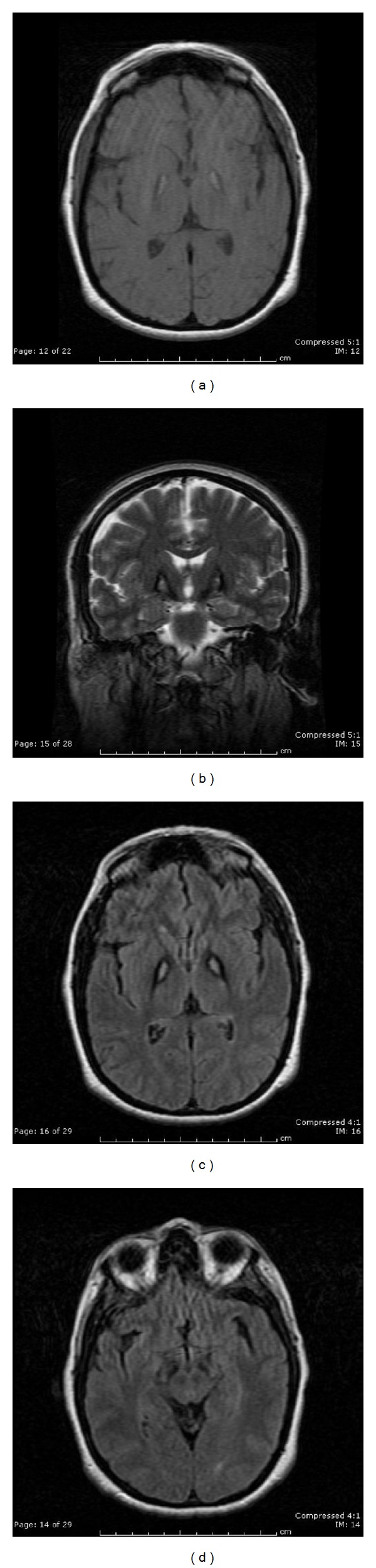
(a) Axial T1 weighted, (b) coronal T2 weighted, and (c) axial fluid attenuation inversion recovery MRI showing low signal intensity in the bilateral globus pallidus with a medial area of signal intensity, presenting the classic “eye-of-the-tiger” sign. (d) Axial fluid attenuation inversion recovery MRI shows bilateral low signal intensity in the substantia nigra pars reticulata.

A 50-year-old woman was referred for neurological evaluation with a two-year history of progressive dysarthria and dysphagia. Two years after the onset of symptoms she was no longer able to sing in church, her speech was difficult to understand, she had difficulty eating, and she complained of throat pain. There was no history of head trauma, no family history of movement disorders, and no history of consanguinity. On examination the patient had involuntary twisting movements of the tongue consistent with orolingual dystonia. Her laboratory workup included normal serum copper, ceruloplasmin, and ferritin levels and no acanthocytes were found on a blood smear. Initial neuroimaging showed no abnormalities. The patient was tried on trihexyphenidyl, diazepam, and carbidopa/levodopa all of which offered minimal relief. Botulinum toxin A injections were effective in treating the orolingual dystonia to a point that allowed her to eat and sing with minimal difficulty for two-month periods. Three years after the initial symptoms she developed blepharospasm and cervical dystonia followed a year later by the appearance of progressive and symmetrical Parkinsonism. Examination also revealed mild cognitive impairment. Due to her progressive symptoms, a follow-up brain MRI was performed and found to show bilateral and symmetrical T2 weighted hypodensities in the globus pallidus with a medial area of hyperintensity consistent with the “eye of the tiger” sign (see Figure 1). This new imaging finding prompted a referral for genetic testing at the age of 54 which revealed a known homozygous pathogenic mutations (881A>T/p.N294I) confirming the diagnosis of PKAN.

## 3. Discussion

Neurodegeneration with brain iron accumulation (NBIA) is an umbrella term for a group of disorders that present with a progressive extrapyramidal syndrome associated with abnormal iron accumulation in the brain, especially the basal ganglia. The main syndrome among the NBIA disorders is PKAN which accounts for more than half the cases of NBIA [3].

Based on clinical features, PKAN can be classified (Table 1) into (a) classic PKAN, with onset in the first decade and a fairly rapid and progressive course leading to loss of independence ten to fifteen years after onset, or (b) atypical PKAN with onset in the second or third decade and a slower disease course that may last 40 years [3]. In both forms, focal dystonias are a common presenting and prominent feature. Children with the classic form often present with a clumsy gait due to limb dystonia. About two thirds of children with classic PKAN also develop pigmentary retinopathy early in the disease which may cause blindness [3]. As the disease progresses, corticospinal tract signs such as spasticity, hyper-reflexia, and extensor plantar toes become supportive in the diagnosis. Orolingual dystonia, leading to dysarthria and dysphagia, can be seen in both forms but is a common presenting feature in atypical patients. Psychiatric symptoms are common in the atypical form and can often predate any motor features and may present as depression, anxiety, emotional lability, obsessive compulsive disorder, or psychosis [4–6]. Progressive cognitive impairment occurs concurrently in both forms. A limited number of reports of PANK2 mutation positive late adult-onset cases (see Table 2) have been published [5, 7–10]. As far as we are aware, our patient is the oldest onset case with the mutation that has been reported. 

The majority of cases of PKAN have a characteristic MR imaging finding known as the eye-of-the-tiger sign (see Figure 1). Abnormal iron accumulation is seen as diffuse and bilateral low T2 weighted signal intensity in the globus pallidus (internal and external segments) surrounding a central area of high T2 weighted signal intensity in the anteromedial globus pallidus corresponding to neuronal loss and gliosis and producing the image of an eye-of-the-tiger [11]. MRI abnormalities may be found early in mutation positive patients [12] but sometimes can lag behind clinical symptoms and, as in our described case, not be seen on initial imaging [13]. The central T2 hyperintensity may be transient and fade with time while evidence of iron accumulation seen as diffuse low T2 signal in the globus pallidus appears later and does not fade [12]. The eye-of-the-tiger was previously considered to have a one-to-one correlation with the presence of a positive PANK2 mutation but more recently several PANK2 negative eye-of-the-tiger cases have been reported [14–17] most of which have been late onset or atypical cases. Therefore, evidence of iron accumulation in the brain may be seen as an indication for genetic testing.

PKAN is an autosomal recessive disorder caused by mutations in the pantothenate kinase 2 (PANK2) gene on chromosome 20 [18]. Numerous mutations in the PANK2 gene have been identified [3]. Homozygous null mutations (resulting in protein truncation) result in classic early onset disease with rapid progression and missense mutations that likely result in partial enzyme function have been associated with atypical late onset disease and slower progression [3, 14]. The mechanism by which PANK2 gene mutations cause abnormal iron accumulation and neurodegeneration is unclear. PANK2 is mainly targeted to the mitochondria [19, 20] and its protein product catalyzes the phosphorylation of pantothenate (vitamin B5) to phosphopantothenate, the first and rate limiting step of coenzyme A biosynthesis. Coenzyme A is an essential cofactor in several metabolic pathways including the citric acid cycle, steroid and heme biosynthesis, amino acid metabolism, and beta-oxidation of fatty acids. Metabolic profiling of patients with PKAN shows mitochondrial dysfunction with an elevated lactate/pyruvate ratio and a deficiency of fatty acids necessary for cellular membrane synthesis [21]. Elevated levels of cysteine, which normally conjugates with phosphopantothenate, have also been observed in PKAN patients [22] and may chelate iron and result in free radical production. Animal models of PANK2 deficiency, PANK2 knock-out mice [23], and a drosophila fruit fly model [24] have failed to reproduce the clinical syndrome seen in humans. In contrast, both Pank2 mutant and wild type mice fed pantothenate (vitamin B5) deficient diets resulted in a progressive movement disorder in wild type mice and an early death in PANK2 mutant mice indicating the importance of pantothenate metabolism in PKAN [25].

Current treatment for all NBIA disorders consists of symptomatic relief. Anticholinergic medications, such as trihexyphenidyl and benztropine, help reduce rigidity, dystonia, and tremor. Baclofen, both orally and intrathecally, is helpful in treating spasticity. Dopaminergic medications may help if there is coexistent Parkinsonism. Benzodiazepines may be useful in the treatment of chorea, tremor, and spasticity. No comparative data exists on the efficacy of these agents. Because dystonia is a prominent feature, injections of Botulinum toxin A may be the most efficacious treatment. Deep brain stimulation (DBS) of the globus pallidus may provide some relief. Several case reports and case series have shown improvements in speech, writing, walking, and global scales of motor function [26, 27] and a single case report has shown long-term benefit [28]. Deferiprone, an iron chelating agent that crosses the blood brain barrier, was studied in a small unblinded pilot trial in four patients with PKAN and showed decreased iron accumulation on MRI in 2 patients and mild to moderate motor function improvement in three patients [29]. Another second small pilot trial using deferiprone in nine PKAN patients demonstrated significant reduction in iron accumulation on MRI but no significant clinical improvement or improvement in quality of life [30]. Further studies using deferiprone are planned.

## 4. Conclusion

We report the case of a woman who developed orolingual dystonia at the age of 48 followed by the onset of blepharospasm, cervical dystonia, Parkinsonism, and mild cognitive impairment. MRI demonstrated the diagnostic “eye-of-the-tiger” sign and genetic testing confirmed a homozygous missense mutation consistent with the diagnosis of PKAN. PKAN is a rare genetic disorder most commonly seen in childhood and adolescence but it should be considered in the differential diagnosis of adult patients with a history of a progressive extrapyramidal syndrome. As the radiographic findings in this disease are quite characteristic, genetic testing should be performed if the MRI shows evidence of iron accumulation. Optimal treatment strategies are not known, and at the current time therapies should be directed at the specific manifestations of the disease. 

## Figures and Tables

**Table 1:** Clinical presentation of PKAN.

Features	Typical PKAN	Atypical PKAN
Onset	First decade	Second or third decade

Features	Gait impairment, focal dystonia, pyramidal dysfunction, pigmentary retinopathy, and cognitive impairment	Psychiatric symptoms, focal dystonia, ± parkinsonism or chorea, cognitive impairment, late gait dysfunction

Progression	Rapid progression Periods of stability interspersed with periods of rapid progression. Loss of ambulation occurs 10 to 15 years after onset	Slower progression Loss of ambulation after 15 to 40 years after onset

Imaging	Eye-of-the-tiger	Eye-of-the-tiger

**Table 2:** Published reports of late adult-onset atypical PKAN.

Reference	Age of onset	Clinical	Eye-of-the- Tiger	PANK2 mutation
Vasconcelos et al. 2003 [7]	36	Dysarthria, tongue atrophy	Yes	Yes
Antonini et al. 2006 [8]	30	Choreoathetosis, postural tremor, personality changes, and paranoia	Yes	Yes
Seo et al. 2009 [9]	35	Parkinsonism	Yes	Yes
Aggarwal et al. 2010 [10]	37	Postural/action tremor	Yes	Yes
del Valle-López et al. 2011 [5]	30	Acute psychosis, clumsiness, and frequent falls	Yes	Yes
